# Medical and Periodontal Clinical Parameters in Patients at Different Levels of Chronic Renal Failure

**DOI:** 10.1155/2017/9858073

**Published:** 2017-04-03

**Authors:** Caroline Perozini, Gilson Fernandes Ruivo, Lucilene Hernandes Ricardo, Larissa Avance Pavesi, Yeon Jung Kim, Debora Pallos

**Affiliations:** ^1^Department of Periodontics, University of Taubaté, Taubaté, SP, Brazil; ^2^Department of Nephrology, University of Taubaté, Taubaté, SP, Brazil; ^3^Department of Implantology and Periodontics, University of Santo Amaro, São Paulo, SP, Brazil

## Abstract

*Aim*. To assess the clinical periodontal and medical parameters in patients with chronic renal failure (CRF) at different levels of renal disease.* Background*. CRF is a progressive and irreversible loss of renal function associated with a decline in the glomerular filtration rate. Periodontal disease is a destructive inflammatory disease affecting periodontal tissues that shows high prevalence in patients with CRF.* Materials and Methods*. 102 CRF patients were included and divided into an early stage group (EG), predialysis group (PDG), and hemodialysis group (HDG). The medical parameters were taken from the patients' records.* Results*. Periodontal clinical condition differed among the CRF groups. Clinical attachment loss was greater in the HDG and PDG group compared to the EG (*p* = 0.0364); the same was observed in the Plaque Index (*p* = 0.0296); the others periodontal parameters did not show any differences. Ferritin levels were significantly higher in the HDG when compared to the EG and PGD (*p* < 0.0001), and fibrinogen was higher in PDG compared with the others (*p* < 0.0001); the triglycerides also showed higher values in the HDG compared with the other groups (*p* < 0.0001).* Conclusion*. The patients with renal involvement should have a multidisciplinary approach to an improvement in their oral and systemic health.

## 1. Introduction 

Chronic renal failure (CRF) is defined as a progressive decrease of renal function associated with reduced glomerular filtration rate. The most common causes of CRF are diabetes mellitus, glomerulonephritis, and chronic hypertension. The clinical signs and symptoms of CRF are dependent on the stage of disease, affect most body systems, and are collectively called uremia. The treatment of this disease includes changes in diet and fluid restriction in order to accommodate the reduced excretion capacity of the kidneys. Despite these treatments, some patients progress to the final stage of the disease, requiring dialysis (hemodialysis and/or peritoneal dialysis) and kidney transplantation [1–3].

Patients in end stage renal disease can develop clinical complications, such as chronic inflammatory diseases, which can be due to a functional defect in the immune system throughout the progress of CRF and also to the continuous nonspecific stimulation of immune cells as a consequence of the extended exposure to dialysis membranes filters during the course of dialysis support. Uremic toxins can also induce an inflammatory process, involving lymphocytes, proinflammatory cytokines, and oxidative stress disorders, which if associated with malnutrition, can be a marker of higher mortality [4–9].

Periodontal disease is a chronic inflammatory condition of the supporting dental tissues, resulting from the infection and interaction of specific bacteria with constituents of the host immune response. Its progression involves the formation of periodontal pockets and the destruction of the collagen structures of the periodontium and alveolar bone. At the most advanced stage of periodontal disease, it is possible to see mobility and premature loss of teeth [10, 11].

The impact of periodontal disease on systemic health is relevant, since it involves a framework of systemic inflammation, stimulating the development and progression of atherosclerosis, as well as promoting difficulties in controlling diseases such as diabetes mellitus, lung disease (cancer and chronic obstructive pulmonary disease), osteoporosis, and renal insufficiency [12, 13].

The existence of possible periodontal disease or any change in oral health may represent a risk to patients on hemodialysis (HD), who are extremely susceptible to complications. These findings are significant for the population in HD, since the elevation of serum markers of inflammation, such as CRP, has been reported as a predictor of cardiovascular mortality in these patients [14, 15]. There are many sources of inflammation in patients on HD. However, with the high incidence of periodontal disease in the general population and the possibility of increased incidence and severity in patients in hemodialysis, periodontal disease can be a source of inflammatory products in systemic diseases, which could be reduced with effective periodontal treatment [7, 16–18].

Inflammation and/or infection in patients with chronic renal disease (CRD) may alter its progression, as indicated by worsening laboratory parameters and the development of signs and symptoms that require dialysis support. Furthermore, some studies have shown a high mortality rate in patients on hemodialysis who had advanced periodontal disease [19, 20].

Studies in the literature that assess the oral conditions in patients with CRD have shown contradictory results. Therefore, this study aimed to evaluate and compare the clinical periodontal conditions in patients with CRD at different stages.

## 2. Materials and Methods 

For this cross-sectional study, we analyzed 102 individuals between 24 and 80 years old (54.74 ± 13.01) who were previously evaluated at the Clinic of Nephrology, University Hospital of Taubaté, Taubaté Institute of Nephrology (INEFRO), in the State Center for Treatment of Kidney Disease and the Paraíba Valley Clinic Nefrovale Pindamonhangaba, SP. Recruitment took place between February 2008 and June 2009.

Dental and medical histories were collected from each participant, and they also underwent a clinical examination that was conducted in the Clinical Dentistry Department of Periodontology, University of Taubaté (UNITAU). The participants were informed about the purpose and methodology of the study and signed a consent form that had been previously approved by the Ethics Committee of the University of Taubaté (UNITAU), Ref: 0485/07.

All patients were identified by a code, so that only the researchers had access to their information. The subjects were divided into three groups according to their CRD stages.

The guidelines developed by the National Kidney Foundation's Kidney Disease and Outcomes Quality Initiative (K/DOQI) [2] defined five stages of CRF on the basis of different ranges of GFR: stage 1, GFR > 90 mL/min/1.73 m^2^; stage 2, GFR 60 to 89 mL/min/1.73 m^2^; stage 3, GFR 30 to 59 mL/min/1.73 m^2^; stage 4, GFR 15 to 29 mL/min/1.73 m^2^; and stage 5, GFR < 15 mL/min/1.73 m^2^.

Subjects were initially divided into three groups that corresponded to their stage of CRD, according to the guidelines of the National Kidney Foundation [2]:

- 44 individuals in the early stages of CRD (1-2), which was considered the early group (EG)
- 30 individuals in the predialysis stage (moderate/severe) of CRF—stages 3-4 (PDG)
- 28 individuals in the terminal stage (dialysis)—stage 5 (HDG)

Exclusion criteria included the following: having received periodontal treatment in the last six months; antibiotic therapy in the 72 h prior to the periodontal examination; and being seropositive for HIV, hepatitis C virus (HCV), or hepatitis B virus (HBV).

Laboratory data were obtained from the medical records of patients who were followed up for renal disease in the same week of the periodontal examination. In cases in which data were unavailable, blood samples were collected for laboratory analysis during the week of the periodontal examination.

The following laboratory parameters were analyzed: CRP, fibrinogen, ferritin, triglycerides, and creatinine. With the data obtained in laboratory tests, the estimated creatinine clearance was determined using the equation proposed by Cockcroft/Gault [21].

The clinical periodontal examination was performed with a manual periodontal probe (PCPUNC 15 Hu-Friedy Co., Inc., Chicago, IL) on all teeth, excluding the third molars. The following parameters were analyzed:

- Probing Depth (PD) was measured by a single examiner at six sites per tooth, three points for vestibular site (mesiobuccal, buccal, and distobuccal), and three points for lingual (mesiolingual, lingual, and distolingual), with a manual Williams type periodontal probe. The PD was measured from the free gingival margin to the base of the periodontal pocket.
- The clinical attachment loss (CAL) was obtained from all examined sites by measuring the distance from the cementoenamel junction (CEJ) to the gingival margin (GM) and adding the PD measurement: CAL = PD + (JEC MG).
- The gingival condition of subjects was assessed using the Gingival Index [22].
- Oral hygiene was assessed by the Plaque Index [23].
- Another parameter was number of missing teeth.

According to the diagnosis established after the periodontal examination, each individual was treated as needed. The treatment was performed in the Clinic of Undergraduate and Postgraduate Periodontics, Department of Dentistry, UNITAU.

Patients were clinically evaluated by a previously trained and calibrated periodontist. Of the sample, 10% were examined twice for each of the clinically evaluated criteria to obtain the intraexaminer reliability, as measured by Kappa (between 0.8 and 1.0). The examiner of the periodontal parameters was blind to the medical status of the patient.

The comparison between the mean values among groups was analyzed using Student's* t*-test for the parametric groups and the Mann–Whitney test for nonparametric groups. In cases in which there was a significant difference (*p* < 0.05) between groups, post hoc comparisons between pairs were conducted using the Tukey-Kramer test. Also the analyses between the groups were performed with ANOVA and Kruskal-Wallis test. Analyses of this study were carried out using the statistical program GraphPad Prism version 4.0 (GraphPad Software Inc., San Diego, CA USA). The unit of analysis used in this study was the average of each parameter per patient.

## 3. Results

The groups were divided according to chronic renal condition. The PDG group exhibited a mean age that was higher than the patients of the EG (*p* = 0.0162) and HDG (*p* < 0.0001) (Table 1). The periodontal parameters showed that the CAL of the PGD and HDG was significantly different from that of the early group (*p* = 0.029 and *p* = 0.0348). The PGD and HDG group showed greater amount of Plaque Index (PI) with statistical significance, and the Gingival Index was the same in the three groups. There were no other statistical differences observed between the groups (Table 2).

On the topic of markers of systemic inflammation, ferritin levels were significantly lower in the EG when compared to those with CRD (PDG and HDG) and in the PDG when compared to the HDG (Table 3). The concentration of triglycerides remained higher in groups with renal disease PDG and HDG. The concentrations of fibrinogen were significantly higher in the PDG, with statistical difference between the EG and HDG. The CRP showed grater levels in the PDG group but with no statistical difference compared with the others. Higher creatinine levels were observed in the HDG compared to the EG and PDG (Table 3).

## 4. Discussion

The present study evaluated the periodontal status of 102 patients who were subdivided according to their renal conditions. Systemic markers related to inflammation were compared in those groups. The results showed that the CAL and PI of the predialysis group and hemodialysis group were greater than those of the early stage group. The number of missing teeth was similar in the three groups. The other periodontal parameters did not show any differences between the groups according to the status of their renal disease. When the medical parameters were analyzed, increased creatine levels were found in the HDG and PDG when compared with the EG. The levels of ferritin and triglycerides were greater in the HDG compared with the other groups. This similarity was not observed for the levels of CRP. Greater levels of fibrinogen were found in the PDG group compared with the other two groups.

Fibrinogen is an independent cardiovascular risk factor. Other acute phase proteins, such as fibrinogen and IL-6, are also directly related to vascular disease. Many patients with chronic kidney disease have elevated serum fibrinogen and thereby have a direct correlation with inflammation, malnutrition, and increased mortality from coronary artery disease [5].

Patients with chronic renal failure shows a progressive decrease in renal function, which may lead to the development of uremic syndrome, characterized by pre- or proinflammatory state that causes immunodeficiency due to the increase of toxic substances in the blood stream. These patients are more likely to develop chronic inflammation, and their condition has been considered to be a nontraditional risk predictor of cardiovascular morbidity and mortality, especially in patients who are terminally ill from CRF [7, 9, 20].

Periodontal diseases are chronic inflammatory processes that begin with microbial infection accompanied by the destruction of periodontal tissues mediated by the host. The bacterial accumulation on the tooth surface is essential for the initiation and progression of periodontal disease. Cells responsible for immune processes, such as neutrophils, initiate the host response against invading microorganisms and periodontal pathogens. Although patients with CRF present oral changes such as gingival overgrowth, dry mouth, increased formation of dental calculus, and changes in bone metabolism, the effects of this systemic condition on periodontal health are not clear. Therefore, this study evaluated the periodontal health of 102 patients, divided into groups according to their renal function.

Several studies have attempted to demonstrate a relationship between periodontal disease and renal disease, but the results were inconsistent [24–29].

One explanation for this diversity of results could be supported by the classification of periodontal disease used in each study. Currently, there is considerable controversy in classifying periodontal disease. In a systematic review published on its different classifications, the authors did not establish a final consensus on what categories and criteria would be the most appropriate. According to the authors, studies with periodontal disease are complicated by the diversity of methodologies and definitions used [30]. This does not occur with kidney disease because the patient is classified with respect to the degree of renal dysfunction using the equations for calculating the estimated creatinine clearance using simplified methods and more specific formulas, such as the Cockcroft/Gault equation [21].

Although there are several studies in the literature that compare periodontal disease with renal failure, most studies have compared patients on dialysis (hemodialysis and peritoneal dialysis) to a control group [24–26, 28, 31–33] and in some cases include a predialysis group [27, 29, 34, 35]. Only the study by Garcez et al. [36] assesses the oral health status in a group with the IRC in its initial stage (stage 2 CRD) by comparing it to a control group. As in our study, the authors noted that although the test group presented serum creatinine levels that were significantly higher than the control group, they were within the normal range according to the parameters, and there were no differences in the oral health conditions of these patients.

The predialysis (PDG) and hemodialysis groups (HDG) exhibited CALs and PI that were significantly greater than those of the EG; however, the other periodontal parameters showed no differences. The results are in agreement with some studies, which compared only the oral condition of patients in end stage renal disease to that of controls [24, 28, 31, 32]. However, the results of this study differed from those of Bayraktar et al. [26] which found higher GI in patients with chronic renal failure.

Moreover another study observed that the PI, GI, bleeding on probing, and CAL were higher in the hemodialysis group compared to predialysis patients, and both groups exhibited higher values than the control group [35]. However, the periodontal conditions were more severe in healthy controls with periodontitis. The authors did not examine the laboratory tests of control groups, and the predialysis group was composed of individuals who were between phases 2 and 5 in the IRC. In a similar study, the authors found statistical differences between the periodontal parameters in chronic renal failure (predialysis and dialysis) patients and controls [27]. However, the population studied by the authors was younger, and the employed classification criterion differs from that of this study. Patients were considered to be predialysis patients when they had a creatinine clearance less than 30 mL/min/1.73 m^2^; this is equivalent to patients with stage 4 CRD. Individuals at this stage have a greater degree of systemic inflammation than individuals in the third phase.

CRP levels were higher in the PDG group, but they were not significantly different from the other groups. Although this marker is considered one of the main factors related to the inflammatory process, in which elevated CRP levels are associated with mortality and cardiovascular disease in CRD patients, the results in the literature are contradictory [37–39]. Others studies noted the relationship between this marker and the periodontal inflammatory process [20, 40–42]. However, the study by Kshirsagar et al. [19] found no such association.

Fibrinogen is also considered to be a marker of inflammatory activity, either by itself or in combination with other markers, such as CRP, especially in the assessment of coronary ischemia. Some studies have shown this association between serum fibrinogen, renal disease, and azotemia. With the progression of renal disease, uremia is associated with the systemic inflammatory response, with a reduction in their values but without normalization of their values after the onset of support dialysis, as contact with dialyzer membranes may also perpetuate the systemic inflammatory reaction, and not all of the toxins and inflammatory cytokines are fully debugged [43]. In this study, the results showed that serum fibrinogen values among patients in the predialysis group were increased compared to the control group and the hemodialysis group, suggesting a higher inflammatory activity in this group [44]. These findings correlate with those in the literature, since the predialysis group exhibited a higher stage of inflammation, which was similar to findings in which fibrinogen was also observed for the CRP results.

A meta-analysis released by the Fibrinogen Studies Collaboration corroborated previously established information, showing a positive correlation between the plasma levels of fibrinogen and the risk of coronary heart disease, cerebrovascular, and other vascular complications in healthy adults [43]. With regard to heart disease, Danesh et al. [45] had previously reported this finding, showing that this condition was associated with higher CRP levels and changes in leukocyte count and serum albumin. This information has a great impact on renal disease, as cardiovascular disease, especially ischemic disease, is the major cause of mortality among nephropathic patients, particularly among patients undergoing hemodialysis.

Other significant results were found: the highest values of triglycerides were found in the predialysis and hemodialysis groups, despite the fact that a distribution of patients with diabetes mellitus, hypertension, and smoking was observed in all studied groups of patients. Hypertriglyceridemia is associated with the cardiovascular risk for atherosclerosis. Currently, hyperuricemia has also been included as an independent risk factor for cardiovascular disease.

Clinical periodontal parameters were greater in the test group compared to the early group. Health professionals should carefully consider CRD in order to establish an interdisciplinary relationship to improve the oral and systemic health of those patients.

## Figures and Tables

**Table 1:** Demographic data of the study population according to the groups.

Groups	EG	PDG	HDG
*N*	44	30	28
Age	52.29 ± 11.92^a^	62.75 ± 11.26^b^	49.40 ± 11.86^a^
Gender (%F)	75.0	50.0	41.7
Hypertension (%)	52.63	80.00	100.00
Diabetes (%)	40.0	30.0	16.7

The same letters represent no statistical difference *p* < 0.01.

**Table 2:** Distribution and comparison of the periodontal parameters according to study groups (mean ± standard deviation).

	EG	PDG	HDG	*p* value
*N*	44	30	28	
PD (mm)	2.62 ± 0.65^a^	2.88 ± 1.09^a^	2.83 ± 0.96^a^	0.6842
CAL (mm)	3.29 ± 1.41^a^	4.30 ± 1.65^b^	4.05 ± 2.04^b^	0.0364
PI	1.61 ± 0.53^a^	1.85 ± 0.53^b^	1.78 ± 0.51^b^	0.0296
GI	1.45 ± 0.55^a^	1.60 ± 0.50^a^	1.61 ± 0.44^a^	0.2785
MT	11.23 ± 8.04^a^	13.77 ± 7.42^a^	11.43 ± 7.55^a^	0.3448

EG—early stage group; PDG—predialysis groups; HDG—hemodialysis group; MT—missing teeth; PD—probing depth; CAL—clinical attachment loss; PI—Plaque Index; GI—Gingival Index; the same letters represent no statistical difference.

**Table 3:** Laboratory parameters of the patients according to study groups.

Groups	Reference value	EG	PDG	HDG	*p* value
*N*		44	30	28	
CRP	<3.0 mg/L	3.4 ± 3.4^a^	4.6 ± 3.5^a^	3.5 ± 1.3^a^	0.1190
Fibrinogen	175–400 mg/dL	274.3 ± 67.1^a^	317.9 ± 45.75^b^	262.2 ± 35.8^a^	<0.0001
Ferritin	22 to 322 ng/mL	134.3 ± 39.6^a^	218.1 ± 145.8^b^	1004.0 ± 952.1^c^	<0.0001
Triglycerides	<150 mg/dL	151.5 ± 67.3^a^	167.7 ± 55.2^a^	206.9 ± 48.3^b^	<0.0001

Creatinine	F-0.6 to 1.2 mg/dL	0.96 ± 0.31^a^	1.36 ± 0.3^b^	9.73 ± 3.63^c^	<0.0001
	M-0.7 to 1.3 mg/dL				

EG—early stage group; PDG—predialysis groups; HDG—hemodialysis group; CPR—C-reactive protein; the same letters represent no statistical difference.
